# Equine Adipose-Derived Mesenchymal Stromal Cells Release Extracellular Vesicles Enclosing Different Subsets of Small RNAs

**DOI:** 10.1155/2019/4957806

**Published:** 2019-03-18

**Authors:** Stefano Capomaccio, Katia Cappelli, Cinzia Bazzucchi, Mauro Coletti, Rodolfo Gialletti, Franco Moriconi, Fabrizio Passamonti, Marco Pepe, Stefano Petrini, Samanta Mecocci, Maurizio Silvestrelli, Luisa Pascucci

**Affiliations:** ^1^Dipartimento di Medicina Veterinaria, Università degli Studi di Perugia, Via San Costanzo, 4, 06126 Perugia, Italy; ^2^Centro di Ricerca sul Cavallo Sportivo (CRCS), Università degli Studi di Perugia, Italy; ^3^Istituto Zooprofilattico Sperimentale dell'Umbria e delle Marche, Italy

## Abstract

**Background:**

Equine adipose-derived mesenchymal stromal cells (e-AdMSC) exhibit attractive proregenerative properties strongly related to the delivery of extracellular vesicles (EVs) that enclose different kinds of molecules including RNAs. In this study, we investigated small RNA content of EVs produced by e-AdMSC with the aim of speculating on their possible biological role.

**Methods:**

EVs were obtained by ultracentrifugation of the conditioned medium of e-AdMSC of 4 subjects. Transmission electron microscopy and scanning electron microscopy were performed to assess their size and nanostructure. RNA was isolated, enriched for small RNAs (<200 nt), and sequenced by Illumina technology. After bioinformatic analysis with state-of-the-art pipelines for short sequences, mapped reads were used to describe EV RNA cargo, reporting classes, and abundances. Enrichment analyses were performed to infer involved pathways and functional categories.

**Results:**

Electron microscopy showed the presence of vesicles ranging in size from 30 to 300 nm and expressing typical markers. RNA analysis revealed that ribosomal RNA was the most abundant fraction, followed by small nucleolar RNAs (snoRNAs, 13.67%). Miscellaneous RNA (misc_RNA) reached 4.57% of the total where Y RNA, RNaseP, and vault RNA represented the main categories. miRNAs were sequenced at a lower level (3.51%) as well as protein-coding genes (1.33%). Pathway analyses on the protein-coding fraction revealed a significant enrichment for the “ribosome” pathway followed by “oxidative phosphorylation.” Gene Ontology analysis showed enrichment for terms like “extracellular exosome,” “organelle envelope,” “RNA binding,” and “small molecule metabolic process.” The miRNA target pathway analysis revealed the presence of “signaling pathways regulating pluripotency of stem cells” coherent with the source of the samples.

**Conclusion:**

We herein demonstrated that e-AdMSC release EVs enclosing different subsets of small RNAs that potentially regulate a number of biological processes. These findings shed light on the role of EVs in the context of MSC biology.

## 1. Background

Mesenchymal stromal cells (MSCs) have risen great interest due to their attractive biological features extensively investigated in human and veterinary regenerative medicine as well as in immune and cancer therapy. It has been demonstrated that MSCs constitutively produce extracellular vesicles (EVs) that are involved in the cell-to-cell transfer of biomolecules [1]. On the basis of their size and biogenesis, EVs have been classified in (i) *microvesicles* or *shedding vesicles* or *ectosomes* (MV) ranging from 100 to 1000 nm and delivered through the outward budding of the plasma membrane and (ii) *exosomes* (EX), 40-100 nm-sized vesicles generated from the endosomal compartment through the inward budding of the outer membrane of multivesicular bodies (MVBs) and their fusion with the plasma membrane [2]. It has been demonstrated that EVs can induce huge changes in the surrounding environment and modify the behavior of target cells by two recognized mechanisms: the transfer of functional proteins and the delivery of RNAs that induce a reprogramming of target cells. Both these mechanisms mainly depend upon EVs' entry into the recipient cells. In other cases, EVs' surface can trigger signaling through interaction with receptors on the cell surface without entry. The transfer of subcellular component or part of them (for example, mitochondria) is another recognized mechanism of action of EVs [3].

An increasing number of scientific evidence seems to demonstrate that EVs display, *in vitro*, similar biological properties as their parental cell counterpart such as promotion of proliferation [4], prevention of apoptosis [5], modulation of the immune response [6, 7], suppression of fibrosis, and promotion of angiogenesis [8–10]. Additionally, MSC-derived EVs exhibited therapeutic effectiveness in different conditions *in vivo* such as graft-versus-host disease, neurite outgrowth, angiogenesis, myocardial ischemia/reperfusion injury, and acute kidney injury [1]. In some of these conditions, EVs appeared even more effective than parental cells themselves, possibly as a result of specific molecule enrichment in their cargo. RNA was initially not considered a mediator of intercellular communication because of its instability and rapid degradation by endogenous RNAses. Recent studies reported that noncoding RNAs, piRNA, snRNA, snoRNA, and tRNAs as well as miRNAs packaged in EVs, were also found in body fluids *in vivo* [11, 12]. Besides representing a promising tool for clinical applications, such as liquid biopsies, this observation highlights the role of vesicle-associated RNAs as “signaling molecules” in cell-to-cell communication [13]. Vesicle-associated RNAs have great advantages over intercellular communication mediated by soluble factors in that EVs could transport many messages at once and control simultaneously functionally related genes determining a complex and fine control of target cells [14].

Despite the rapidly increasing number of reports regarding the therapeutic effects of MSC-EVs, detailed investigations identifying the cargo molecule(s) responsible for these effects are still limited [15–17].

In a paper by Baglio et al. [18], the full small RNA landscape of human adipose and bone marrow MSCs and their corresponding exosomes was characterized. The authors demonstrated that small RNA component in MSC-derived exosomes does not reflect the cellular content and is characterized by a few miRNAs that are overrepresented in comparison to the cells of origin. In addition, they demonstrated that, besides the most studied miRNAs, other noncoding transcripts characterize the vesicular content.

Regarding veterinary species, Eirin et al. [19] studied the RNA cargo of EVs derived from porcine adipose tissue MSCs observing that they are selectively enriched for distinct classes of RNAs and contain low levels of miRNA.

Concerning the equine species, only a few recent papers demonstrated that MSCs from different tissue sources constitutively produce EVs and that they are partly responsible for cells' paracrine activity [9, 20–22]. However, the contribution of RNA content in determining these actions remains to be explored.

In this study, we proposed an in-depth analysis of small RNAs associated to the total pool of EVs released by equine adipose-derived MSC in order to speculate on their possible contribution to the biological behavior of parental cells and lay the foundation for future functional studies.

## 2. Methods

### 2.1. Aim, Design, and Setting of the Study

We firstly evaluated morphological features of EVs harvested from the conditioned medium (CM) of equine adipose-derived MSC (e-AdMSC) by transmission electron microscopy (TEM) and scanning (SEM) electron microscopy. Then, we investigated the RNA content of e-AdMSC with the aim to understand the mechanisms of their biological action. In particular, we sequenced with NGS approach their small RNA content (<200 nt) in order to hypothesize how the RNA content could potentially alter different recipient cell populations inferring the mostly affected gene and pathways on target cells.

### 2.2. MSC Culture and EV isolation

Subcutaneous adipose tissue samples were obtained from the abdominal wall of 4 horses, three months to four years old, referred to OVUD (Ospedale Veterinario Universitario Didattico, University of Perugia) for minor surgeries. Detailed description of age, sex, and breed is available in the Supplementary Material and in the BioSample platform (SRR7142093-96). An informed consent was requested from the owners, and the sampling procedure was approved by the Ethics and Welfare Committee of the University of Perugia (protocol number 2012-034). Samples were rapidly brought to the laboratory in phosphate-buffered saline (PBS), with 200 U/ml penicillin, 200 mg/ml streptomycin, and 12.5 mg/ml amphotericin B (Merck, Darmstadt, Germany). Tissue samples were washed twice in PBS with antibiotics and antimycotic and were then submitted to mechanic fragmentation and digestion with 0.075% collagenase type I (Worthington Biochemical Corp., Lakewood, NJ) at 37°C for 45 minutes. The homogenate was centrifuged at 600g for 10 minutes to separate the lipid fraction that was discarded. The pellet containing the stromal vascular fraction was seeded in tissue culture flask and incubated at 37°C with 5% CO_2_ with DMEM low glucose (Dulbecco's modified Eagle Medium; Gibco, Gaithersburg, MD) supplemented with 10% fetal bovine serum (FBS), 100 U/ml penicillin, and 100 mg/ml streptomycin. Adherent cells were maintained in culture with complete medium and passed 1 : 3 when reached 80% of confluence. Cells at passage 3 were characterized for their multipotent differentiation potential and for the expression of CD90, CD44, and CD73 as previously described in the literature [23, 24]. About 50 × 10^6^ cells at passage 3 for each subject were seeded in T75 flasks to obtain EVs. After 24 h from seeding, the medium was replaced with DMEM supplemented with 0.5% BSA and with 100 U/ml penicillin and 100 mg/ml streptomycin. EVs were isolated as previously described with modifications [9]. Briefly, MSC-conditioned medium was collected after 48 h of culture in serum-free conditions and centrifuged at 300×g for 10 minutes to remove dead cells and then at 2000×g for 25 minutes to remove floating debris. The supernatant was then transferred to Ultra-Clear tubes (Beckman Coulter Inc., Woerden, the Netherlands) and centrifuged at 100,000×g for 80 minutes at 4°C in a SW70Ti rotor (Beckman Coulter Inc., Woerden, the Netherlands). The pellet was gently washed with PBS, centrifuged again at 100,000×g for 1 h, and finally suspended in PBS. A small volume of vesicle suspension was used for transmission electron microscopy (TEM) and scanning (SEM) electron microscopy analysis in order to evaluate morphological features of isolated EVs. The residual part was snap frozen in liquid nitrogen and stored at −80°C for small RNA extraction.

### 2.3. Electron Microscopy

For TEM analysis, several drops of EV suspension (20 *μ*l/drop) were placed on Parafilm. Formvar-coated nickel grids (Electron Microscopy Sciences, Hatfield, Pa, USA) were gently placed on the top of each drop for 60 minutes at room temperature, the coated side of the grid facing the suspension. After washing on phosphate buffer (PB) 0.1 M pH 7.3, grids were fixed with 2.5% glutaraldehyde (Fluka, St. Louis, MO, USA) for 5 minutes, washed in distilled water, and contrasted with 2% aqueous uranyl acetate. Finally, the grids were washed in distilled water, air dried, and observed under a Philips EM 208 transmission electron microscope equipped with a digital camera (University Centre for Electron Microscopy (CUME) Perugia).

For SEM analysis, EVs were allowed to adhere to formvar-coated nickel grids and fixed with 2.5% glutaraldehyde as described for TEM. The grids were attached on metal stubs, coated with chrome to a thickness of 10 nm, and examined with a ZEISS LEO 1525 (Nanomaterials Laboratory, University of Perugia).

### 2.4. Immunoelectron Microscopy

Formvar-coated nickel grids with adherent EVs (see the previous section) were transferred for 10 minutes on single drops of 1% PBS-BSA (blocking buffer) to block unspecific sites. The grids were then incubated overnight at room temperature with mouse monoclonal anti-CD90 antibody (VMRD Inc., WA, USA) diluted 1 : 20 and rabbit anti-flotillin1 polyclonal antibody (Bioss Antibodies Inc., MA, USA) diluted 1 : 100 in blocking buffer. After several washes in PBS to remove the excess of antibody, the grids were incubated for 1 hour at room temperature, respectively, with goat anti-mouse secondary antibody and goat anti-rabbit, gold-conjugated secondary antibody (Jackson ImmunoResearch Laboratories, PA, USA) diluted 1 : 40 in PBS with 1% BSA. Grids were finally washed in PBS, counterstained with 2% aqueous uranyl acetate for 5 min, washed with distilled water, and examined with a Philips EM 208 transmission electron microscope equipped with a digital camera (University Centre for Electron Microscopy (CUME) Perugia).

### 2.5. RNA Extraction and Sequencing

EV pellets from previous steps were used for RNA extraction. *mirVana*™ miRNA Isolation Kit (Ambion–Life Technologies, MA, USA) was used to extract total RNA enriching for the fraction of small RNAs (<200 nt) that are known to be mostly represented in EVs. RNA quantity and quality were evaluated using NanoDrop 2000 spectrophotometer (Thermo Fisher Scientific, Waltham, MA, USA), Qubit 2.0 Fluorometer (Life Technologies, MA, USA) and by microfluidic electrophoresis on Bioanalyzer 2100 (Agilent Technologies, Santa Clara, CA, 95051 USA).

Library preparation was carried out following the TruSeq Small RNA Library Prep Kit specifications for the four samples recruited in the study. Massive parallel sequencing was carried out on an Illumina HiSeq 4000 platform. Individual samples are labeled in the paper as EC and a progressive number.

### 2.6. Bioinformatic Analysis

RAW reads from the sequencer were checked for quality and trimmed from unwanted sequences (adapters) using FastQC (https://www.bioinformatics.babraham.ac.uk/projects/fastqc) and Trimmomatic v. 0.33 [25], respectively.

After this step, the resulted reads were mapped using STAR v.2.5.0b guided by the Ensembl v.90 transcript annotation downloaded from the UCSC table browser, on the *Equus caballus* reference genome (equcab2). To avoid bias in describing the RNA cargo using uniquely mapped reads on the genome, aligned reads were classified as counts on genes with the software *featureCounts* using the Ensembl coordinates [26]. Operations on genome coordinates were performed with *bedtools* and in-house built scripts.

To assess the most abundant transcripts, RPKM (reads per kilobase per million) were calculated with edgeR package [27] averaging the expression of the four libraries.

A transcript was considered expressed when overcame the 10 RPKM threshold. Protein-coding genes expressed at RPKM >10 were queried into STRING-DB to search for pathway and GO (ene Ontology) term enrichment. This step was performed also querying against the *Homo sapiens* annotation.

GO analysis on miRNA targets was performed with mirPath v.3 from DIANA Tools utilizing default parameters and *Homo sapiens* naming. Unique names for miRNA were obtained converting IDs with miRNAme converter [28].

## 3. Results

**Figure 1:**
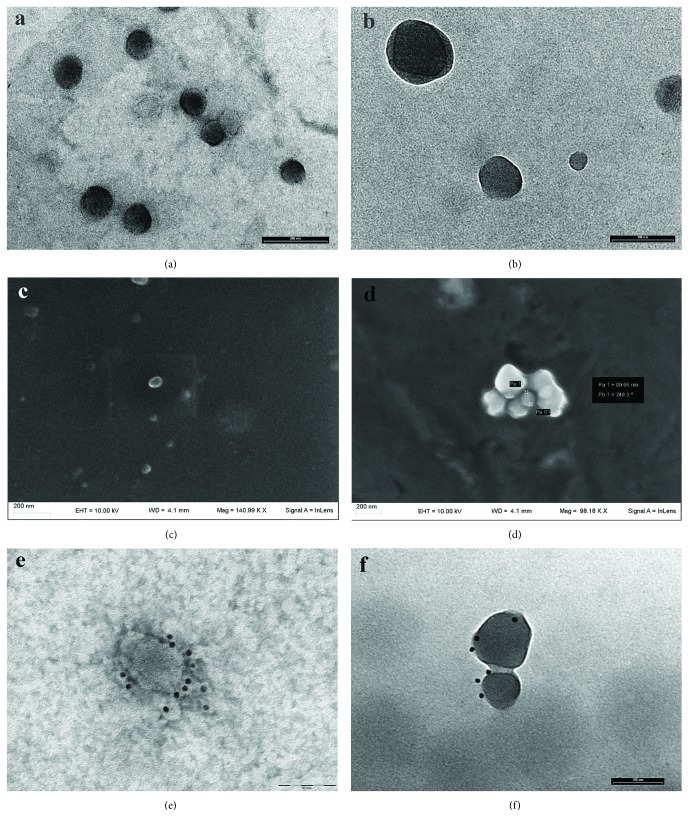
Electron micrographs depicting EVs isolated by e-AdMSC supernatants. (a) Electron micrograph showing round-shaped EVs. TEM, scale bar, 200 nm. (b) High magnification micrograph showing several vesicles. TEM, scale bar, 100 nm. (c) The figure represents isolated EVs observed by SEM. Scale bar, 200 nm. (d) The figure depicts a small aggregate of EVs observed by SEM. Scale bar, 200 nm (e, f) Immunoelectron microscopy. CD90-positive EVs (e) and flotillin-1-positive EVs (f). Scale bar, 100 nm.

By electron microscopy analysis, we observed a population of single vesicles ranging in size from 30 to 300 nm and showing, at TEM, a peripheral limiting membrane (Figures 1(a)–1(d)). The vesicles expressed CD90 and flotillin-1 that are considered, respectively, markers of MSC-derived shedding vesicles and exosomes (Figures 1(e)–1(f)). The use of serum-free culture medium made it possible to exclude any potential contamination by FBS-derived EVs.

Sequencing produced about 145 million read pairs that were used as input for the bioinformatics pipeline. On average, 69% of the cleaned reads were uniquely aligned to the equine reference genome (equcab2): detailed statistics on the quality check and mapping process are reported in Table 1. Due to the nature of the sample and the shortness of the transcript sequences, only uniquely mapped reads (UMR) were used to further evaluate the EV cargo.

The Ensembl annotation (ver 90) served as a guide for classifying UMRs into transcripts and classes of transcripts (Table 2).

**Figure 2:**
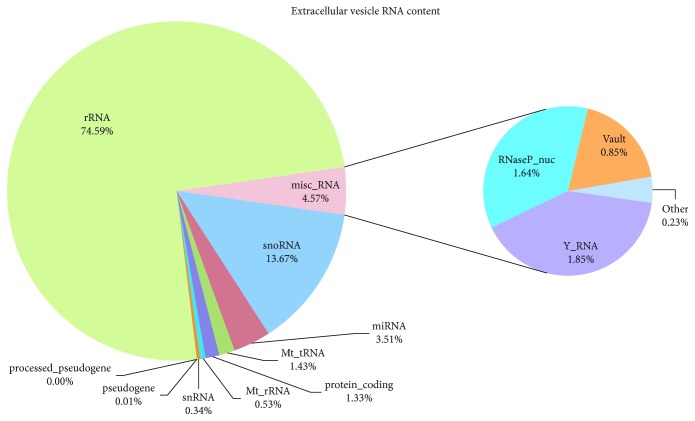
Extracellular vesicle cargo pie chart. Each slice corresponds to one RNA category. Percentage of sequences referred to the UMR is reported.

The annotated fraction of the genome accounted for roughly the 73% of the UMR. Classification of the reads and membership percentage is also depicted in Figure 2.

The great part of the sequences belonged to rRNA, followed by small nucleolar RNAs (snoRNAs, 13.67%), a large conserved group of small noncoding RNAs. Miscellaneous RNA (misc_RNA) reached 4.57% of the total where Y RNA, RNaseP, and vault RNA represented the main categories. miRNAs were sequenced at a lower level (3.51%) as well as protein-coding genes that lowered down to 1.33%. During the classification process, we noticed that some miRNA showed extremely high coverage, so we checked every miRNA that did not have a corresponding gene name. All poorly annotated miRNAs resulted to be dead entries according to miRBase database and were manually reclassified in the correct category to avoid representation bias (see Additional File 1).

To better interpret our results, we decided to set an expression value cut-off in RPKM (reads per kilobase per million base pairs) in order to exclude scarcely transcribed molecules. High variability was observed between samples but this is consistent with previous findings [29].

Features with values lower than 10 RPKM were no further investigated. Five hundred and sixty-seven (567) features/genes overcame the threshold and resulted to be divided as detailed in Table 3.

From these data appear clearly that the most represented features were the snoRNAs with millions of reads assigned (276 records), followed by the protein-coding fraction (154) and miRNA (38). rRNA, while being the most sequenced category, had one of the lowest numbers of individual transcripts. Additional File 1 contains expression values for each considered transcript.

**Figure 3:**
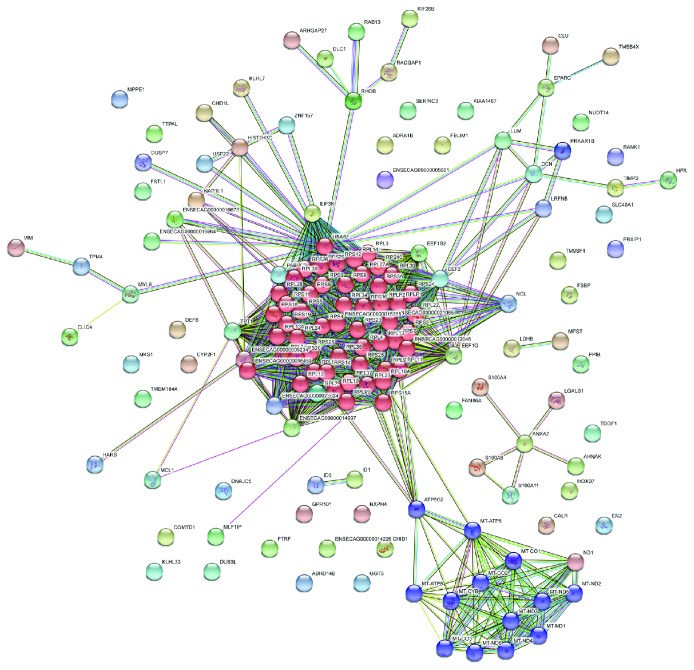
Connection between the protein-coding fraction of the cargo according to SRING v10 analysis. Two KEGG pathways are highlighted: in red, “ribosome”; in violet, “oxidative phosphorylation.”

To assess if some functional enrichment was present in the EV cargo, the one hundred and fifty-four protein-coding genes were investigated using STRING [30], and a clear signature emerged from this group. Pathway analyses on the protein-coding fraction revealed that there was a significant enrichment for the “ribosome” pathway with 53 proteins out of the 154 considered (Figure 3, red-colored cluster), followed by “oxidative phosphorylation,” (Figure 3, violet-colored cluster). GO (Gene Ontology) analysis revealed enrichment for terms like “extracellular exosome” and “organelle envelope” concerning the cellular component vocabulary, “RNA binding” as regards molecular function vocabulary, and “small molecule metabolic process” concerning biological process vocabulary. Full result tables are available in Additional File 2.

**Figure 4:**
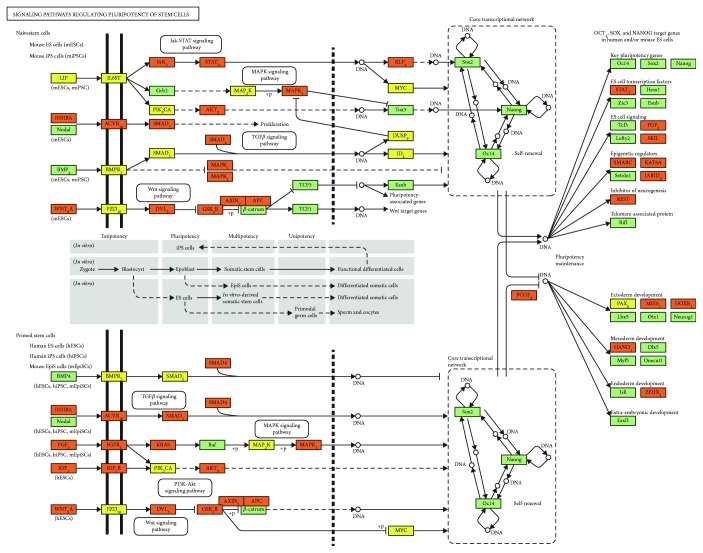
Graphical visualization of the enriched KEGG pathways for miRNA targets: all targets belonging to the pathway are in green squares. Different colors represent the number of hits: gene that is target of 1 miRNA (yellow); gene that is target of more than 1 miRNA (orange).

The miRNA target pathway analysis through mirPath v.3 revealed that there were enriched pathways coherent with the source of the samples: “signaling pathways regulating pluripotency of stem cells” are probably one of the most interesting since it involved almost all miRNAs (24) and a significant amount of gene targets (71). Moreover, this pathway is highly interconnected with other significantly enriched pathways like “TGF-beta signaling,” “MAPK signaling,” “PI3K-Akt signaling,” and “Wnt signaling” (Figure 4). Full results of mirPath v.3 analyses are available in Additional File 3.

## 4. Discussion

In recent years, the use of MSC has proved to be a hopeful tool in the treatment of several pathologic conditions of horses including tendon lesions, articular diseases, and skin wounds [31–33]. Among the different horse tissues employed for MSC collection, adipose tissue is one of the sources of choice, due to the relative ease of access and the number of multipotent cells that can be efficiently collected and expanded *in vitro* [34, 35].

Regardless of species and tissue source, it has been demonstrated that MSC may exert their biological action through the release of EVs containing bioactive molecules of different chemical nature. In particular, the presence of different species of RNA has been described in a number of papers [36]. The presence of RNA inside EVs suggests that, upon internalization by recipient cells, they directly influence their gene expression. EV-associated RNAs display quantitative and qualitative differences in several diseases and have been proposed for use as biomarkers. Furthermore, due to their ability to influence target cell functions and to transport and deliver drugs, they have the potential to be exploited therapeutically [13, 37].

To our knowledge, only a few studies have analyzed RNA content of MSC-EVs by deep sequencing and none of them was conducted in horses. Therefore, this is the first study that outlined small RNA profile in the entire subset of vesicles released by equine MSC under standard culture condition.

We firstly analyzed the morphological features of isolated EVs by electron microscopy and confirmed that all the four samples contained a mixture of shedding vesicles and exosomes. It has not still been completely clarified whether different RNA biotypes share specific enrichment in distinctive subsets of MSC-derived vesicles. Furthermore, most of the common EV isolation methods fail to separate accurately vesicle subpopulations, so that the process of purification of MV or EX may be considered an enrichment rather than a separation [36]. For this reason, the experimental design was conceived in order to purify the entire population of EVs and to recover all the RNA species delivered by e-AdMSC inside EVs.

The use of ultracentrifugation for vesicle separation was chosen in order to achieve the highest recovery, despite a low specificity collection. Considering the nature of conditioned medium and the serum-free cultivation, the main particles that risked to be co-isolated along with MV and EX were apoptotic bodies. Although the serum-free cultivation has not been validated for e-AdMSC nor the effects of serum free conditions on cellular metabolism were determined in this study, trypan blue dye exclusion demonstrated that >98% of the total cells were viable after serum deprivation. Furthermore, the occurrence of apoptotic bodies affecting the results of our study may be considered negligible since no nuclear changes attributable to early or late stages of apoptosis were observed at cell analysis by TEM (data not shown).

As concerns RNA analysis, together with a small fraction of protein-coding transcripts, we identified different types of noncoding RNAs. In particular, beyond ribosomal RNA fraction, a plethora of noncoding RNAs with new regulatory roles as snoRNAs were the most abundant part of the cargo, followed by miRNAs. Below is a more detailed description of most represented groups.

SNORDs are the highly abundant class of ncRNAs and predominantly serve as guides for the chemical modification of ribosomal RNA; however, about one-half of known snoRNAs shows no sequence complementarity toward other ncRNAs and thus are orphan, suggesting not yet discovered additional functions.

In our EVs, we found 276 features accounting to SNORDs, most orphans, representing nearly 14% of the entire cargo.

Orphan SNORDs can play roles in cellular regulation processes such as alternative splicing, microRNA production, and cholesterol traffic. In addition, they are implicated in several pathological conditions such as neurodevelopmental disorders and oncogenesis, with unfortunately no clues about their mechanism of action [38, 39]. snoRNA genes are often located in the intronic region of genes encoding proteins involved in ribosome synthesis or translation. They are also located in intergenic regions, ORFs of protein-coding genes and UTRs [40].

Intriguingly, a large part of snoRNA genes are subjected to genomic imprinting, an epigenetic phenomenon that restricts gene expression to only one chromosome and a possible role in the evolution or mechanism of imprinted loci has been suggested [41].

In our EV RNA cargo, there are many examples of snoRNA genes subjected to imprinting phenomena in the human genome [42] (see Additional File 1).

Moreover, snoRNAs can have miRNA like function; in silico analyses revealed that putatively snoRNA-derived miRNA-like fragments appear in different organisms. The processed snoRNAs, indeed, have been implicated in pre-miRNA processing and in controlling gene expression [39].

However, the true dual function of sno/miRNA molecules is not yet elucidated: they are probably themselves under the control of other regulation layers that ensure the appropriate balance of the different species originating from these molecules in different cell types [43, 44].

The link between snoRNA and miRNA is even tighter if we consider that retrotransposition events may have played a major role in the mobility and function of both [45].

Another known noncanonical function of SNORDs is their influence on alternative exon selection. Alternative splicing is a critical step in the maturation of the vast majority of RNAs and also a key regulator for gene expression. Alternative splicing, indeed, can indirectly regulate transcript abundance because of alternative exon configuration can introduce frameshifts or stop codons leading to nonsense mediated decay into the pre-mRNA [46].

Even in this case, the SNORDs known to have a sure regulatory function by influence on alternative splicing are represented in our cargo with SNORD27 [43] and others (see Additional File 1).

Functional intercellular transfer of miRNAs through EVs has been extensively described [37] and has been proved to be extremely influenced by the cells from which they derive [29]. Even if in our analyses only 28 annotated features fall in known miRNAs (see Additional File 1), they may provide clues as to the function of EV progenitor cells as potential effects on angiogenesis, cell differentiation, and proliferation that fit well with the already described effects of MSCs.

Pro-angiomiR-30, which represents one of the most abundant miRNA of e-AdMSC-EV cargo, promotes angiogenesis by targeting different regulators in angiogenic signaling pathways. miR-30, carried by exosomes, plays an important role in MSC-mediated angiogenesis and has been demonstrated that exosomes with inhibited expression of this miRNA lead to reduced angiogenesis [47].

Also, mir-221 and let-7 have been described to have proangiogenic effects as may target the receptors of angiogenic factors. In particular, miR-221 was identified as a regulator of c-Kit expression controlling the ability of endothelial cells to form new capillaries upon vascular endothelial growth factor (VEGF) stimulation. Furthermore, let-7 members participate in angiogenesis by regulating the expression of the antiangiogenic factor tissue inhibitor of metalloproteinase- (TIMP-) 1 [48].

VEGF, in addition, regulates the expression of several other miRNAs, including cluster miR-17–92 that include mir-93 contained in our cargo. VEGF levels enhance also neovascularization correlated with downregulation of antiangiogenic thrombospondin-1 (Tsp1) that are predicted targets for repression by the miR-17–92 miRNA cluster [49].

e-AdMSC-EVs also contain miR-100, known to be enriched in MSC-derived exosomes and associated with the downregulation of VEGF in a time-dependent manner. In addition, the downregulation of VEGF mediated by MSC-derived exosomes can affect the vascular behavior of endothelial cells in vitro; therefore, exosomal transfer of miR-100 may be a novel mechanism underlying the paracrine effects of MSC-derived exosomes and may provide a means by which these vesicles can modulate vascular responses within the microenvironment of cancer cells [50].

A further study showed that miR-125a, enriched in our e-AdMSC-EVs, was enriched also in human adipose-derived MSC exosomes. miR-125a repressed the expression of the angiogenic inhibitor delta-like 4 (DLL4) by targeting its 3′ untranslated region modulating endothelial cell angiogenesis through promoting the formation of endothelial tip cells. Human adipose-derived MSC exosomes indeed can transfer miR-125a to endothelial cells to promote angiogenesis by repressing DLL4 [51].

Another miRNA of AdMSC-EV cargo, miR-93, promotes angiogenesis and cell survival and is strongly expressed in several types of cancer cells in which it acts as oncomiR, regulating tumor growth, cell survival, migration, apoptosis, cell-cycle distribution, and angiogenesis [47, 52–54].

miR-99b, part of our AdMSC-EV cargo, instead, is enriched in endothelial cells differentiated from human embryonic pluripotent stem cells. It was reported that overexpression of this miRNA enhances pluripotent cell differentiation potential and improves their angiogenic activity in a mouse model of limb ischemia [55].

Our miRNA gene target investigation and the subsequent pathway enrichment analysis seem to confirm our speculations about the possible effects of miRNAs' cargo on recipient cells and their involvement in cell function regulations at the autocrine and paracrine level. In fact, KEGG pathways constructed from target genes revealed signaling pathways regulating cell fate, and in particular, proliferation, differentiation, apoptosis, and senescence of stem/progenitor cells with 71 target genes of 24 miRNAs among the most significant (2,77E-05). Other pathways were related to the control of biological functions such as cell growth and inflammation in which MSCs are known to be involved.

Not only many miRNA target genes are coherent with our biological system but it is also clear that most of them are targets of more than one miRNA (Figure 4) [56].

The analysis of the protein-coding gene cargo revealed a clear enrichment signature. Network analysis showed that there was a significant enrichment for the “ribosome” pathway with 53 proteins out of the 154 considered (Figure 3) and 13 proteins that belong to the “oxidative phosphorylation” pathway. Both pathways had extremely low *p* values. GO analysis revealed enrichment for terms related to extracellular content such as “extracellular exosome” or outer membranes with the cellular component (CC) vocabulary. “RNA binding” and “oxidoreductase activity” are among the most represented ones for molecular function whereas “cellular protein metabolic process” and “translation” for biological process vocabulary (see Additional File 2). These data allowed us to hypothesize an activation of “anabolic processes signals” mainly towards protein synthesis and energy metabolism. In particular, the presence of proteins belonging to the respiratory chain or any way peculiar to mitochondrial metabolism leaves us to speculate that a subset of AdMSC-EVs had a mitochondrial origin. Recent studies have demonstrated that proteins and mtDNA are secreted in the form of microvesicles and exosomes by MSCs and by cells derived from different malignancies [57, 58]. Diversely from the intracellular release of mitochondrial derived vesicles that undergo a degradation pathway [59], mitochondrial vesicles released in the extracellular space or fused with exosomal pathway (Mt-EVs) seem to be clearly related to cell-cell communication [58]. Even if this topic still needs to be deeply elucidated, it could be speculated that Mt-EVs are addressed to target cells in order to enhance their mitochondrial function. This issue is particularly interesting in the context of regenerating tissues.

Several papers speculated about the possible presence of distinct RNA species in a different subpopulation of EVs. Besides microvesicles produced by cell shedding, [60] they demonstrated that cells release at least two major kinds of exosomes that may be separated on the basis of differences in migration speed in the sucrose gradient and characterized by different sizes and density: high-density exosomes (HD-exo) and low-density exosomes (LD-exo). Willms and colleagues [60] observed that MVs and LD-exo displayed 18S and 28S ribosomal RNA peaks and small RNAs peak found at ~60 nt. In contrast, HD-exo did not contain rRNA but contained a broad range of RNA species ranging from 30–150 nt. This indicates a certain heterogeneity within EV RNA content and suggests the existence of subpopulations of exosomes with distinct molecular composition and different effects on recipient cells. It is not surprising, therefore, that the RNA analyzed in this study and derived by the entire population of vesicles produced by AdMSC contains small regulatory RNA as well as fragments of rRNA, as a result of the mixture of MV and EX cargo. Discrimination between subpopulations of EVs could be of great importance for detailed studies on their biology and functions.

Finally, it should be emphasized that the great individual variability between the samples is consistent with previous literature findings observed in different species where a similar degree of divergence between samples has been found. Further studies and a larger sample would be useful to better dissect this issue.

## 5. Conclusion

MSC-derived EVs retain per se a huge therapeutic potential through beneficial properties related to the activation of protein synthesis, cell proliferation, and angiogenesis. The small RNA cargo, characterized for the first time in the equine species via deep sequencing, seems to specifically diffuse this message through a plethora of regulating RNA species and genes that belong to meaningful pathways.

The elucidation of the mechanisms underlying their biological effects needs to be deeply explored in view of a future clinical use.

## Figures and Tables

**Table 1:** Quality-controlled, trimmed, and mapped sequences (pairs).

Sample	Total raw reads	Total clean reads	Uniquely mapped (*n*)	Uniquely mapped (%)
*EC 1*	35,933,150	17,812,049	11,284,218	63.35
*EC 2*	37,226,455	23,933,269	17,354,696	72.51
*EC 3*	32,804,197	23,569,807	17,856,077	75.76
*EC 4*	39,039,549	14,396,847	9,626,487	66.87

**Table 2:** Distribution of reads mapped into known genomic compartment as defined in the Ensembl classification.

	*EC 1*	*EC 2*	*EC 3*	*EC 4*
*miRNA*	575,488	142,383	373,909	149,937
*misc_RNA*	214,930	964,077	290,113	401,141
*Mt_rRNA*	90,057	10,783	20,409	41,973
*Mt_tRNA*	154,033	39,733	101,250	172,063
*processed_pseudogene*	546	304	367	229
*protein_coding*	177,502	75,522	125,678	93,394
*pseudogene*	1,774	815	1,150	973
*rRNA*	3,767,994	10,681,215	12,651,503	5,301,691
*snoRNA*	1,987,323	1,196,097	1,319,036	565,693
*snRNA*	33,811	91,527	12,730	6,173
*Total on known features*	7,003,458	13,202,456	14,896,145	6,733,267

**Table 3:** Number of features at  RPKM > 10 according to the Ensembl annotation.

FeatureType	Number of occurrence
snoRNA	276
protein_coding	154
miRNA	38
rRNA	34
SnRNA	25
Mt_tRNA	20
misc_RNA	12
pseudogene	4
Mt_rRNA	2
processed_pseudogene	2

## Data Availability

Raw sequences are already submitted in SRA and are available upon publication with the following BioProject ID: PRJNA470785.

## Abbreviations

e-AdMSC: Equine adipose-derived mesenchymal stromal cells
MSC: Mesenchymal stromal cells
EV: Extracellular vesicles
ncRNA: Noncoding RNA
MV: Microvesicles
EX: Exosomes.

## Supplementary Materials

Supplementary 1 Additional File 1: gene transcript abundance in RPKM.

Supplementary 2 Additional File 2: Gene Ontology and pathway analysis full results.

Supplementary 3 Additional File 3: pathway analysis of miRNA targets.

Supplementary 4 Additional File 4: bioanalyzer results.

Supplementary 5 Additional File 5: donors' description table.
