# Supplementary material for: Equine Adipose-Derived Mesenchymal Stromal Cells Release Extracellular Vesicles Enclosing Different Subsets of Small RNAs
Source: Stem Cells Int. 2019 Mar 18;2019:4957806. doi: 10.1155/2019/4957806 (PMC6442443; doi:10.1155/2019/4957806)
Supplement: Supplementary 4 — Additional File 4: bioanalyzer results. [file 4957806.f4.pdf]

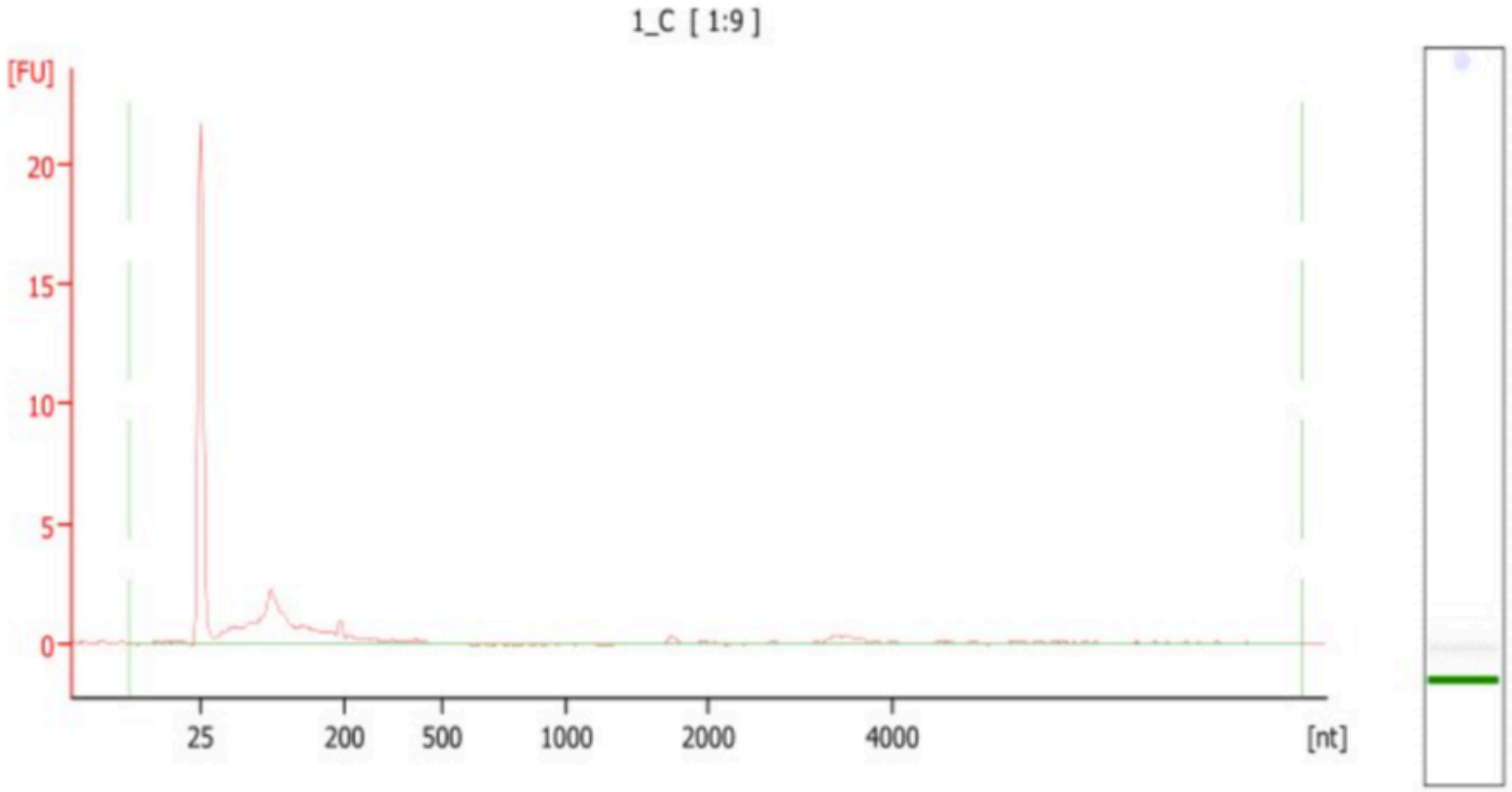

Overall Results for sample 1 : 1_C

RNA Area:	21.4	RNA Integrity Number (RIN):	1.1 (B.02.07)
RNA Concentration:	65 pg/µl	Result Flagging Color:	
rRNA Ratio [28s / 18s]:	0.0	Result Flagging Label:	RIN: 1.10

2_C [1:9]

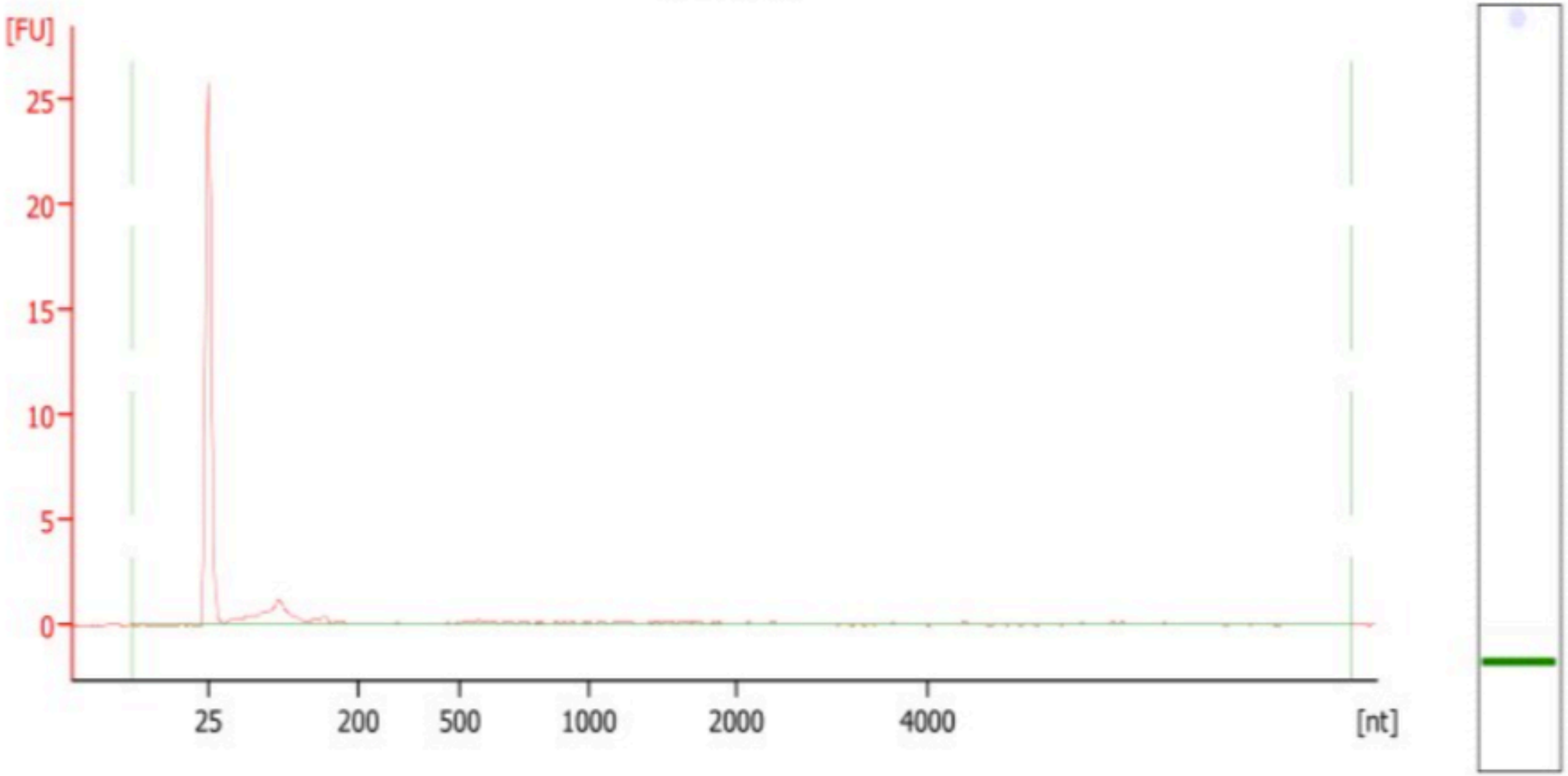

Overall Results for sample 3 : **2_C**

RNA Area:	11.8	RNA Integrity Number (RIN):	1 (B.02.07)
RNA Concentration:	36 pg/µl	Result Flagging Color:	
rRNA Ratio [28s / 18s]:	0.0	Result Flagging Label:	RIN:1

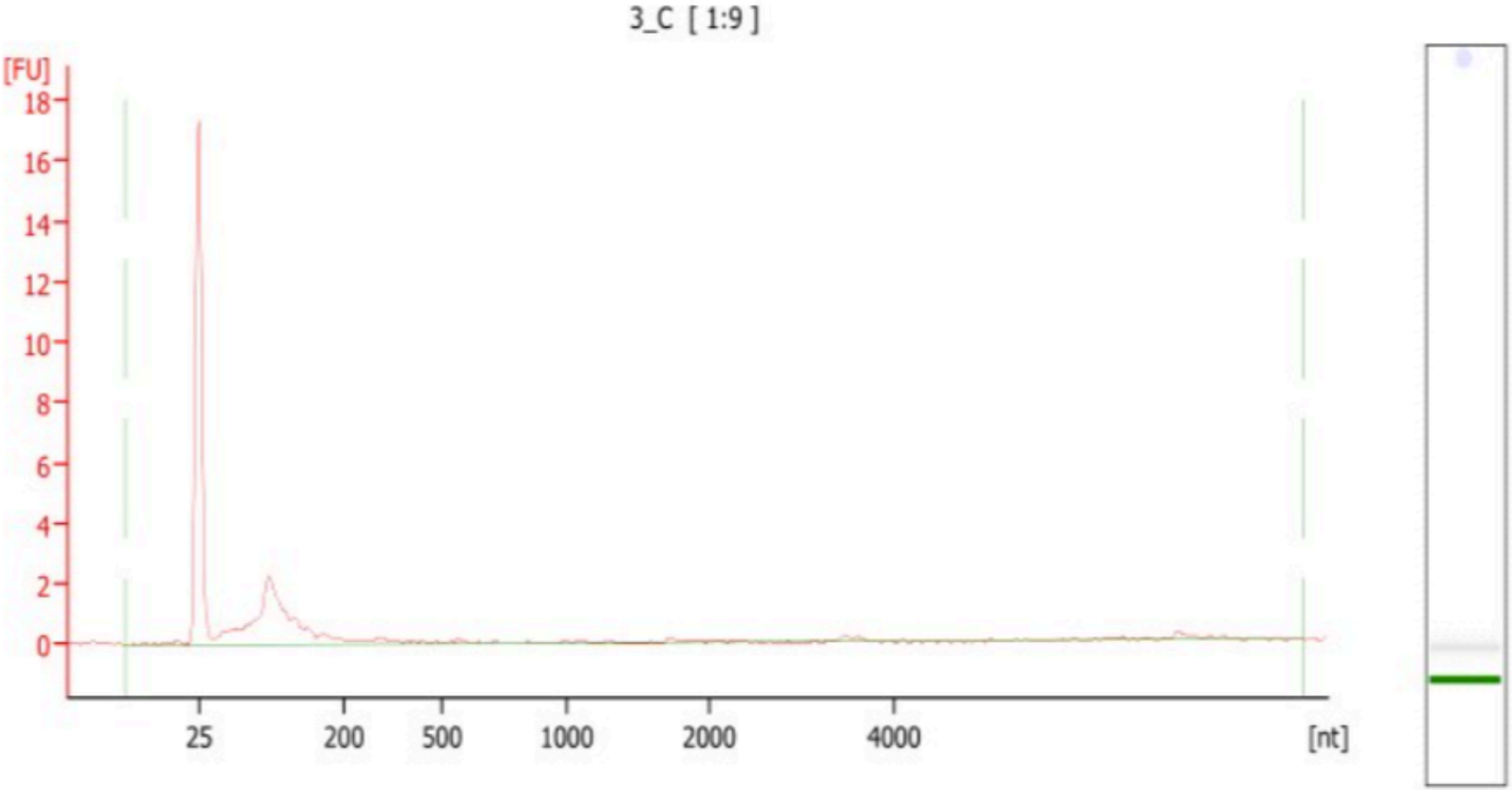

Overall Results for sample 5 : 3_C

RNA Area:	20.7	RNA Integrity Number (RIN):	1.3 (B.02.07)
RNA Concentration:	63 pg/µl	Result Flagging Color:	
rRNA Ratio [28s / 18s]:	0.0	Result Flagging Label:	RIN: 1.30

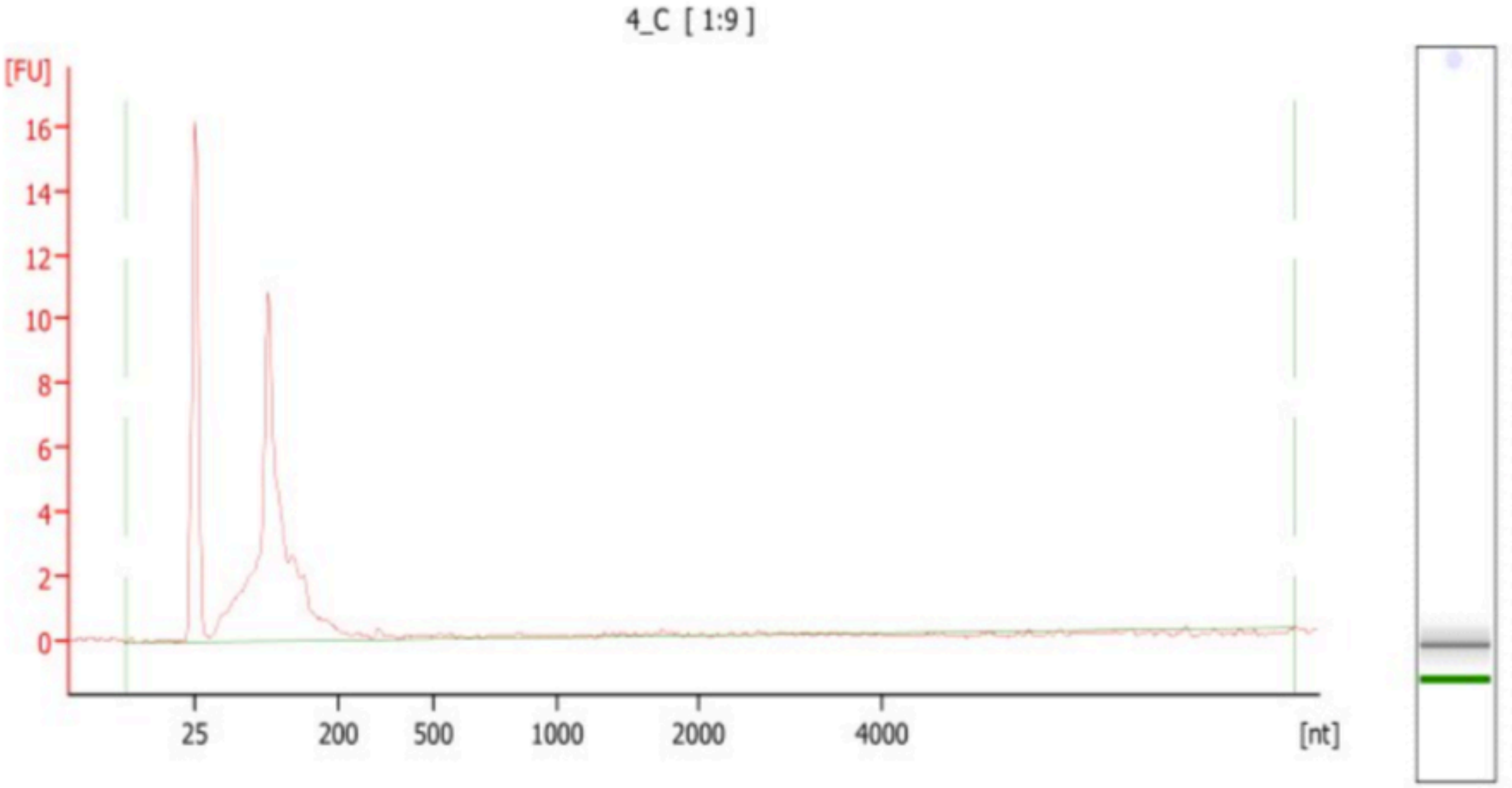

Overall Results for sample 7 : 4_C

RNA Area:	51.6	RNA Integrity Number (RIN):	2.6 (B.02.07)
RNA Concentration:	157 pg/µl	Result Flagging Color:	
rRNA Ratio [28s / 18s]:	0.0	Result Flagging Label:	RIN: 2.60
